# Animal behavior informed by history: Was the Asiatic cheetah an obligate gazelle hunter?

**DOI:** 10.1371/journal.pone.0284593

**Published:** 2023-04-20

**Authors:** Mohammad S. Farhadinia, Bagher Nezami, Ali Ranjbaran, Raul Valdez

**Affiliations:** 1 Durrell Institute of Conservation and Ecology, School of Anthropology and Conservation, University of Kent, Kent, United Kingdom; 2 Department of Biology, University of Oxford, Oxford, United Kingdom; 3 Research Group of Biodiversity and Biosafety, Research Center for Environment and Sustainable Development, Pardisan Park, Tehran, Iran; 4 Journalist, Payam-ma Newspaper, Tehran, Iran; 5 Department of Fish, Wildlife and Conservation Ecology, New Mexico State University, Las Cruces, NM, United States of America; Cheetah Conservation Fund, Namibia University of Science and Technology, NAMIBIA

## Abstract

Understanding key ecological adaptations, such as foraging, when a predator is almost extinct is complex. Nonetheless, that information is vital for the recovery of the persisting individuals. Therefore, reviewing historical, ethnobiological and recent records can assist in exploring the species behavioral ecology. We applied this approach to Asiatic cheetahs (*Acinonyx jubatus venaticus*), which once roamed most west and central Asian countries but now is confined to a few dozens in Iran, at historical (pre-1970) and recent (post-1970) scales. We addressed a widely popular perception that Asiatic cheetahs were subjected to prey shifts from gazelles (*Gazella* spp.) in open plains areas to urial (*Ovis vignei*) in mountains because of gazelle populations declines due to anthropogenic influences. We also quantified recent prey choice of Asiatic cheetahs and their behavioral plasticity in foraging different prey species types. Although ethnobiological and historical records suggested that gazelle species were the main prey for cheetahs across their Asian range. However, urial were also commonly reported to be hunted by cheetahs across their historical Asian range, showing that the predation on mountain ungulates is not an emerging hunting behavior in Asiatic cheetahs. We found spatiotemporal plasticity in recent hunting behavior of cheetahs with selective predation on adult urial males. There was temporal overlap in hunting times for plains dwelling versus mountain ungulates, albeit with some minor differences with morning mostly for gazelles while the predation on mountain ungulates was predominantly post-midday. We provided three management implications for the recovery and restoration of cheetahs in Asia. Our work highlighted the importance of historical studies in informing the behavioral ecology of rare species.

## Introduction

Understanding the foraging ecology of Asiatic cheetahs (*Acinonyx jubatus venaticus*), with a remnant population of a few dozen in the wild is difficult. Nonetheless, it is needed to inform spatial planning for conservation measures, i.e., what prey and which habitat to be prioritized for protection, and potentially restoring the extinct range of cheetahs in Asia [1]. As a consequence of anthropogenic pressures, the Asiatic cheetahs are reported to have experienced a shift in their habitat use from flat areas to more unsuitable habitats, such as hilly and mountain habitats, which may have induced also diet changes from gazelles (*Gazella* spp.) to mountain ungulates, notably urial (*Ovis vignei*) [2–4]. To evaluate this assumption, we ask what was the prey base of Asiatic cheetahs in historical times when prey density and diversity was higher?

Historically, this is not a new controversy for Asiatic cheetahs, as the question of prey selection has been argued for at least 50 years. The historical range of Asiatic cheetahs largely overlapped with three gazelle species: goitered gazelle (*G*. *subgutturosa*), chinkara (*G*. *bennettii*) and blackbuck (*Antilope cervicapra*). Consequently, they were widely noted as the main prey for cheetahs in Asia and their disappearance largely contributed to the widespread decline and localized extirpation of cheetahs across many parts of their Asian range, such as in central Asia [5, 6], Arabian Peninsula [7] as well as Iran [4, 8–11]. In Iran, where the only extant population persisted, cheetahs appeared to have switched to mountain ungulates such as urial, bezoar goat (*Capra aegagrus*) and mouflon (*O*. *melina*) as their prey because of the scarcity of gazelles [2, 3].

In contrast, some authors reported that Asiatic cheetahs depended on a spectrum of medium-sized ungulates, such as gazelles and urial [6, 12–14]. Thus, the recovery of Asiatic cheetah depends on the protection and management of the prey spectrum, rather than a single prey species [15]. Differences in prey choice can be associated with differences in habitat use for cheetahs as gazelles mainly live in open plains while urial mainly occur in hilly terrains and mountains [16, 17]. Lack of agreement on these issues contributed to a widespread debate among conservation practitioners on the most effective initiatives for the recovery of Asiatic cheetahs [18]. For example, differing opinions arose among conservationists as to whether to prioritize gazelle or urial populations and habitats to restore cheetahs given the limited conservation resources [2–4, 19].

In this paper, we addressed this controversy by reviewing a combination of historical and recent records. We defined 1970 as the division point between historical and recent time periods, which coincides with the last records of Asiatic cheetahs in many of their former range countries, afterwards mainly confined to Iran [5, 20, 21]. We reviewed zoological records and published studies based on faecal samples analysis. Zoological records offer a distinctive perspective on the composition of ecosystems and ecological interactions [22]. However, apart from delineating the species historical ranges [5, 20, 21], they are rarely used to interpret species behavioral ecology across temporal scales which can be used to optimize management interventions in conservation biology. We also applied an ethnobiological approach, defined as the study of the interactions of people and the environment [23] to understand historical ecological knowledge on Asiatic cheetahs related to prey. Finally, we quantified recent foraging ecology of Asiatic cheetahs based on opportunistic sightings made by wildlife conservation rangers in Iran. Accordingly, we developed three hypotheses related to historical (hypothesis 1) and recent foraging ecology (hypotheses 2 and 3) of Asiatic cheetahs:

First, given the morphological adaptions of cheetah as a highly specialized, cursorial felid that has evolved as a rapid pursuit predator [24, 25], increased heterogeneity in the landscape such as vegetation density [26, 27] or topographic features [28] reduces the speed of cheetah pursuit, and therefore habitat type may affect predation rates. We therefore expected that historical records would reveal that gazelles were the exclusive prey of cheetahs because those prey species mainly live in open plains compared to mountain ungulates such as urial and bezoar goats.

Second, we questioned the determinants of recent prey choice made by Asiatic cheetahs given that their prey species is currently scarcer than historical times. We thus hypothesized that the prey choice is affected by cheetah social structure [29] and season [30, 31].

Finally, we evaluated the role of different prey in the recent diet of cheetahs and how predation upon different types of prey is temporally adjusted by Asiatic cheetahs. [2, 4, 12, 32].

In the light of these findings, we discussed strategies for cheetah conservation and research within an ecosystem dynamics framework. Also, our study is applicable to decision-makers in countries within the former range of Asiatic cheetahs [1] which may plan to reintroduce cheetahs in the future.

## Materials and methods

We reviewed the foraging ecology of Asiatic cheetahs at two historical and recent scales:

### Historical foraging ecology of Asiatic cheetahs

We searched for two types of historical records:

1) Zoological records in west and central Asian countries (1890–1980): We reviewed existing zoological records within the former range of cheetahs in Asia. We consulted only previous works that included original records and excluded those records whose data was based on secondary sources, instead of their own personal observation or data. These records were atlases, field guide, reports, and other documents. We reviewed 30 records, but 11 were excluded because of the lack of acknowledgement of cheetah prey and habitat or language barrier (S1 Table). Therefore, 19 zoological records were included in the current study.

We also looked for historical literatures and diaries which included cheetahs and their prey in Iran. When prey was not noted, we recorded habitat type, if mentioned, as a proxy for prey, i.e., plains were assumed to represent gazelle habitats whereas mountains were considered habitats for urial and bezoar goat. We reviewed eight historical books that were related to hunting and wildlife in Iran (S2 Table). Two books, originally written before the 12^th^ century, were hunting manuals known as "Baznameh" or "Shekarnameh". The other six were written during by Qajar dynasty, which ruled Iran from 1789 to 1925 and recorded as diaries of the royal family during their hunting trips. We only included those records that contained both cheetah and its prey/habitat explicitly.

2) Ethnobiological records (1000–1900): We searched the word “Yuz” (Persian: یوز), meaning cheetah in Farsi, on a website www.ganjoor.net of an online repository of 191 Persian writers and poets, which include 1,407,424 verses of poems. We first evaluated the accuracy of the website search engine by comparing the search results between the website and an independent portable document file (PDF) version of the resource. Our evaluation was based on “the Shahnameh” and “Ghazaliyate Saadi”, two well-known Persian poetry books which confirmed similar results for both online and PDF versions of mentioned books.

We then searched all the verses for the word "Yuz" and only included them if the name of prey was also mentioned. Those possible prey included "Ahoo" (Persian: آهو) for goitered gazelle, "Jebeer" (Persian: جبیر) for chinkara, "Ghorm" or "Ghorman" (Persian: غُرم، غُرمان) and "Mish" (Persian: میش) for urial, "Goor" (Persian: گور) for onager (*Equus hemionus onager*) and, "Pazan" (Persian: پازن) for bezoar goat. We also included the word "Rang" (Persian:رنگ) as a representative for the bezoar goat, following Moein’s Persian Dictionary [32]. We then counted the repetition of each prey species’ name in the poem’s verses separately to illustrate the possible contribution of each species in the cheetah prey spectrum (S3 Table).

### Recent foraging ecology of Asiatic cheetahs

**Fig 1:**
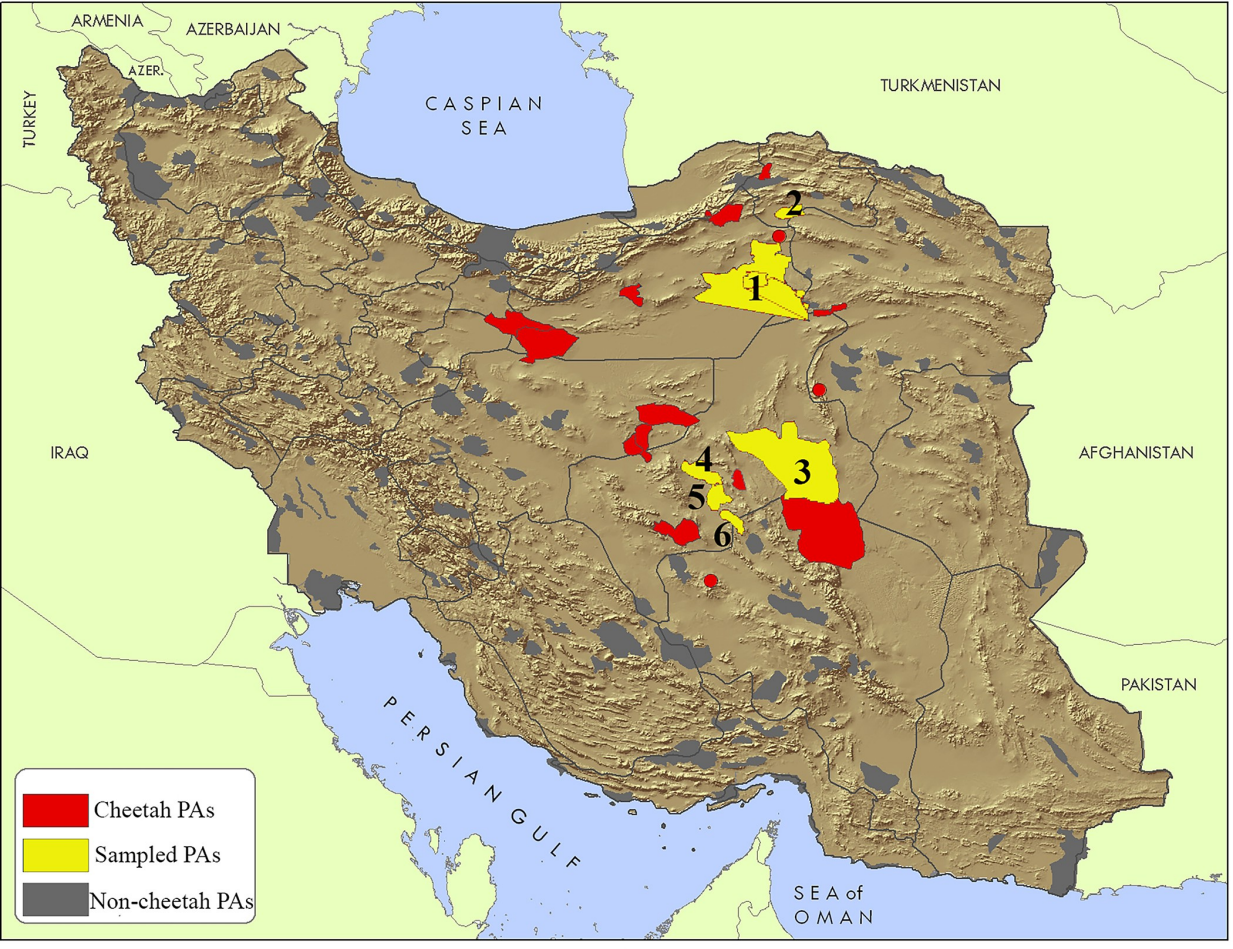
Distribution of the Asiatic cheetah in Iran. Red patches denote to cheetah protected areas confirmed between 2000 and 2020. Yellow areas and their associated numbers represent key cheetah protected areas investigated for faecal samples between 2002 and 2016. 1) Touran, 2) Miandasht, 3) Naybandan, 4) Dareanjir, 5) Bafq and 6) Ariz. The latter three collectively are labelled as Yazd in this study. The map was modified after [33].

Our review of the recent foraging ecology was based on two sources of data post-1970 related to Asiatic cheetahs in Iran:

1) Faecal samples (collected between 2006 and 2017): We compiled a dataset of cheetah faecal samples, collected, and analysed from six cheetah protected areas in Iran (Fig 1). The data were retrieved from published papers in peer-reviewed journals (n = 5). One study was based on DNA barcoding of faecal samples while the rest were based on microscopical identification (S4 Table).

2) Opportunistic sightings (2000–2021): Direct sighting of cheetah predation behavior is extremely rare in Iran. We therefore interviewed wildlife conservation rangers (n = 115) working within the cheetah protected areas to record any sighting of cheetah predation behavior. The data were assigned as “hunting”, i.e., the cheetah was seen while ambushing and chasing the prey or “foraging”, defined as when the cheetah was seen eating the prey. To rule out the occurrence of scavenging, we only included those foraging records in which the kill was examined by the wildlife conservation rangers for canine punctures on the throat or signs of struggling before making the kill. For each sighting, we recorded the date, time, location, number of cheetah, and prey features including species, age, and sex.

### Statistical analysis

We reported descriptive statistics for all historical and recent records. For faecal analysis, we quantified the frequency of occurrence for each prey item, defined as the percentage of total scats in which a food item was found, which is helpful to document rare prey items [34].

To explore the prey choice, we used mixed-effects cumulative link models for the analysis of nominal responses. Multinomial models were fitted using the ‘mixcat’ package [35] in R [36]. Each prey type was treated as a nominal response variable. Predictor variables included season (4 seasons), cheetah social structure and protected area. Cheetah social structure was assigned as solitary (only one individual sighted), family (group of female and cubs) and coalition (multiple adult cheetahs hunting together). We included a random intercept for all models. The significance of terms in the final model was assessed using log-likelihood ratio tests for comparing the goodness of fit between models, following Farhadinia et al. [37]. We also used Fisher’s exact test to test if prey sex was independent of the sexual composition of the prey population, based on adult ungulates killed by cheetahs. We assumed the sex ratio for all ungulates as 40 males per 100 females based on previous studies in similar landscapes in Iran [37, 38].

We also used the ‘overlap’ package to quantify the temporal pattern of cheetah predation behavior [39]. We first split the observation data into two phases of hunting and foraging. We then split the observations of cheetahs during the hunting phase into two categories of prey types, i.e., mountain ungulates (urial and bezoar goat) and plain-dwelling ungulates (goitered gazelle and chinkara). After converting time to radians, a probability density curve was produced for hunting phase. We then quantified the degree of temporal overlap between the two types of prey during hunting phase using the coefficient of overlapping, Δ, where a value of 0 represents no overlap and 1 represents complete overlap. Meredith & Ridout [39] suggested using three variants of the Δ estimator (Δ_1_, Δ_4_, and Δ_5_). When the smaller sample is less than 50, Δ_1_ performed best. The 95% confidence intervals were obtained using 10,000 bootstrap samples. As the coefficient of overlap is a descriptive statistic, Watson’s two-sample *U*^2^ test for circular data was performed using the “circular” package [40] to calculate significance estimates between density curves.

## Results

### Historical foraging ecology of Asiatic cheetahs

In all 19 zoological records that were included in the current study, gazelle species were reported as the main prey for cheetahs across their former range in Asia (Table 1). Equally important, whenever other sympatric medium-sized ungulates existed, they were also reported as cheetah prey in Asian countries. For example, in Iran and the former USSR, both gazelles and urial were reported whereas in the absence of urial within the cheetah range in Iraq, Arabian Peninsula and India, several gazelle species were included as potential cheetah prey. The only exception was Pakistan, where cheetahs formerly inhabited gazelle and urial habitats, but only the former was considered as the cheetah prey, while the urial was never documented as cheetah prey. Large-sized ungulates, such as kulan (*Equus hemionus kulan*) and nilgai (*Boselaphus tragocamelus*) were also considered as occasional prey for cheetahs in the former USSR and India, respectively, but without providing further evidence, such as a specific sighting (Table 1). Similarly, except for Iraq and the Arabian Peninsula, where open plains were reported as the only habitat for Asiatic cheetahs, they used to be found in a variety of habitat types in the rest of their Asian range, from open plains to hills and skirt of mountains (Table 1).

**Table 1:** The zoological records in historic range of cheetahs in Asia (1890–1980) which included data on prey and habitat selection of cheetahs. Latin names of prey species are as the following: Goitered gazelle (*Gazella subgutturosa*), Saiga (*Saiga tatarica*), Urial (*Ovis vignei*), Chinkara (*Gazella bennettii*), kulan (*Equus hemionus*), blackbuck (*Antilope cervicapra*) and nilgai (*Boselaphus tragocamelus*).

Country	Main prey	Habitat type	Reference
Iraq	Gazelle	Desert	[41]
	Gazelle	Plain	[42]
Arabian Peninsula	Gazelle	Open plain	[43]
	Gazelle	Open plains and lowland deserts	[7]
Iran	Gazelle	Desert	[44]
	Goitered gazelle, chinkara and urial	Skirt of mountains	[12]
	Goitered gazelle, chinkara and urial	Foothills and semi-arid plains	[13]
	Goitered gazelle, chinkara and urial	Open plains and low hills	[45]
Turkmenistan	Goitered gazelle, urial and occasionally kulan	Plain and hilly country	[6]
	Ungulates (Goitered gazelle, etc.)	Low or hilly desert	[46]
Kazakhstan	Goitered gazelle, saiga, urial	Plain and hilly country	[6]
Pakistan	Chinkara	Hilly country	[47]
	Goitered gazelle and chinkara	Open plains	[48]
Afghanistan	Goitered gazelle	Open areas	[49]
	Goitered gazelle	Large river basins, clay, and sand biotopes	[50]
India	Blackbuck, chinkara, occasionally nilgai	Open plains and low hills	[51]
	Blackbuck, chinkara, occasionally nilgai		[52]
	Blackbuck, gazelles, deer, and other prey	Low, isolated, rocky hills and surrounding plains	[53]
	Antelope	Low hills bordering the plain	[54]
Jordan and Palestine	Not mentioned	Wooded hills and mountains	[55]

When reviewing historical literatures and diaries about falconry and hunting in Iran, we only found eight books explicitly reporting cheetah prey and habitat (S2 Table). As a result, 16 references to cheetahs were found in these books. The gazelle was the most frequently mentioned prey of cheetahs, followed by urial, hare, onager, bezoar goat, and wild pig *Sus scrofa*, respectively. We also found both open plains and mountains as cheetah habitats in historical times. For example, written in 12 A.D., *Baznameh Nasavi* explained how cheetahs were captured and trained to hunt gazelles in open plains [56]. In contrast, *Masoudi Diary History*, which is devoted to hunting diaries and trips of Mass’oud Mirza Zell-e Soltan (1850–1918 A.D.), the son of Iran’s Qajar King reported that he hunted around 30 cheetahs in his lifetime in open plains or mountains [57].

We obtained 192 ethnobiological records of cheetahs from Iran, such as poems and hunting diaries, spanning between 1000 and 1870 AD. After the exclusion of those verses which did not cite prey species (n = 128), a total of 64 verses were retained mentioning prey species. In total, gazelle was the most frequent prey species (n = 51, 70.0%), followed by urial (n = 13, 18%). Surprisingly, onager was noted 7 times (10.0%; S3 Table).

### Recent foraging ecology of Asiatic cheetahs

**Fig 2:**
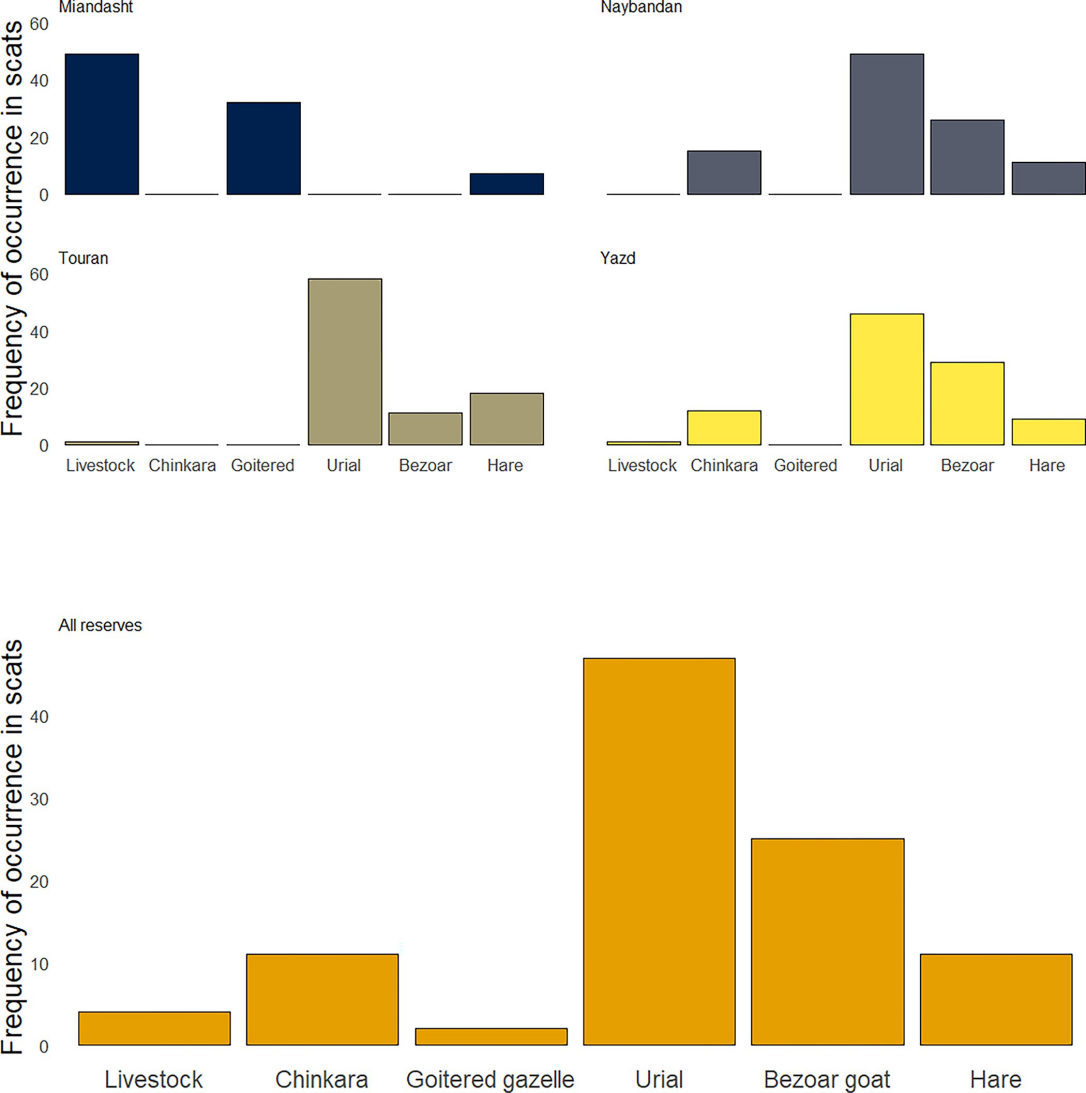
Quantification of different prey species in the diet of Asiatic cheetahs based on five published faecal analyses sampled between 2002 and 2016 across key cheetah protected areas (Yazd, Touran, Miandasht and Naybandan) in Iran [2, 4, 58–60]. Yazd represented three closely located protected areas, including Dareanjir, Bafq and Ariz. Frequency of occurrence is defined as the frequency of that prey item in the total number of faecal samples.

We obtained food habits data analysis of 672 Asiatic cheetah faecal samples from five published papers, sampled between 2002 and 2016, reporting 11 different species killed by Asiatic cheetahs in Iran, from rodents to dromedary camels *Camelus dromedarius* (S4 Table). On average, each faecal sample contained 1.1 prey items. As the dominant wild ungulate in cheetah habitats, urial scored the highest frequency of occurrence (46.6%) in the entire survey period, followed by bezoar goat (24.9%; Fig 2). In contrast, two gazelles existing in cheetah habitats accounted for only 13.2 of the frequency of occurrences of prey items, closely followed by Cape hare *Lepus capensis* (11.0%). Surprisingly, livestock depredation was uncommon, with only 3.6% frequency of occurrence (Fig 2).

**Fig 3:**
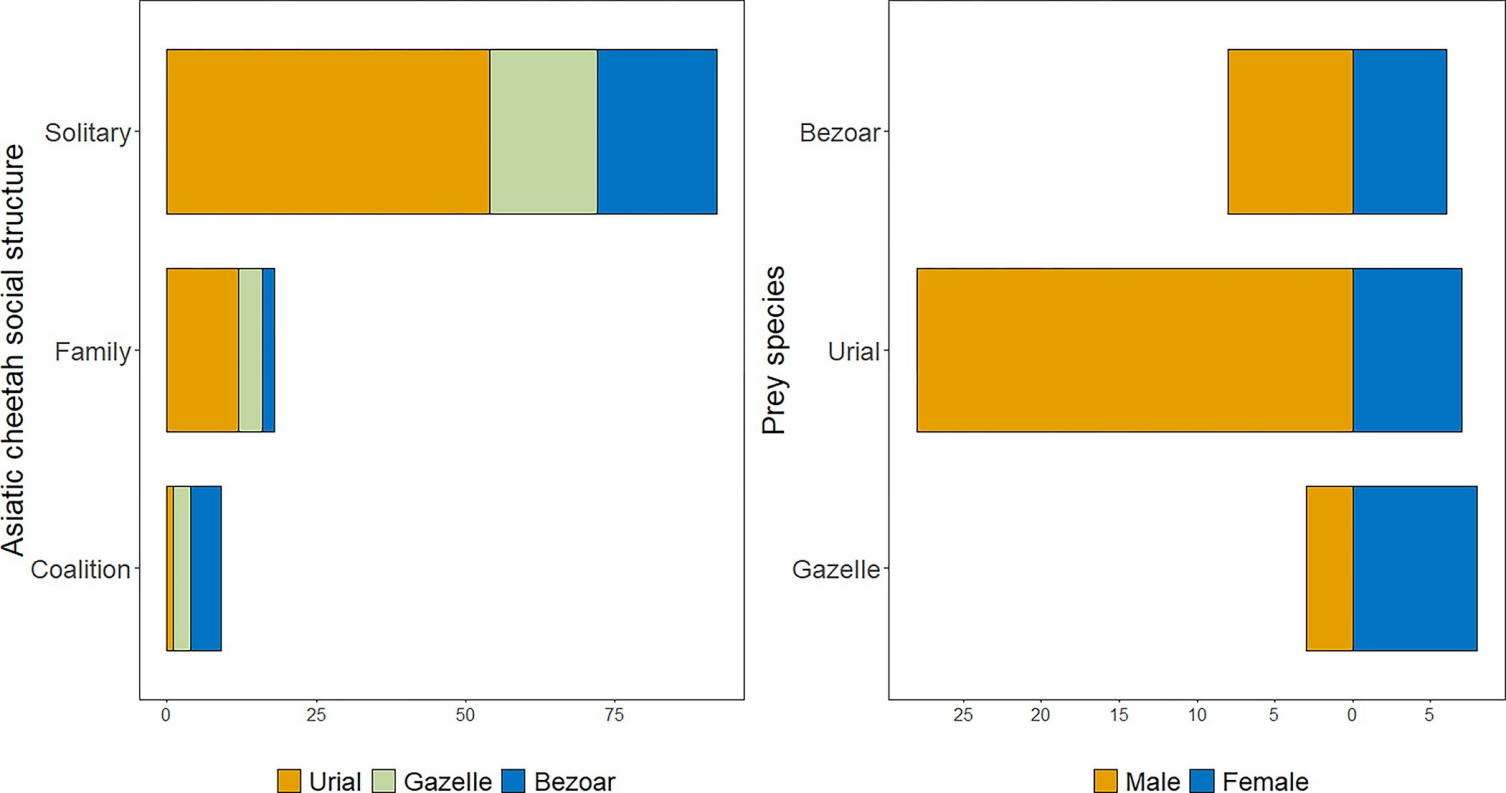
Left) social structure of Asiatic cheetahs in relation to different types of ungulate and right) sex composition of wild ungulates killed by Asiatic cheetahs based on opportunistic sightings made by wildlife conservation rangers between 2000 and 2021 in Iran.

We also obtained a total of 125 predation efforts documented by wildlife conservation rangers between 2000 and 2020 across seven protected areas in Iran (S5 Table). They comprised 43.2% (n = 54) of attempted hunting efforts while the rest represented cheetahs foraging at a kill (56.8%, n = 71). After the exclusion of 11 kills of Cape hare, livestock, or juveniles of wild ungulates, urials represented 58.3% of 60 kills of wild ungulates made by Asiatic cheetahs, which were highly skewed towards males (80.0% rams versus 20.0% ewes; Fig 3). Importantly, Asiatic cheetahs killed both male and female gazelles independent of their abundance (Fisher’s exact test, *P* = 0.99), whereas Asiatic cheetahs killed a preponderance of male urial and bezoar goats which was disproportionate to the ungulates’ highly female-skewed sex ratio in the wild (Fisher’s exact test, *P* < 0.05). Our multinomial models using opportunistic sightings showed that there was no evidence that prey choice was influenced by season, social structure of cheetahs or protected area (Table 2 & Fig 3).

**Table 2:** Results of sequential likelihood ratio tests of multinomial models with random intercept testing the effect of season, social structure, and protected area on prey choice. LR stat. = likelihood ratio statistic (difference of residual deviance); NA = Not Applicable.

Model no.	Explanatory variables	Test	*d*.*f*.	LR stat.	*P*
1	Null	NA	NA	226.7	NA
2	Season	2 versus 1	3	224.7	0.57
3	Social structure	3 versus 1	2	225.5	0.55
4	Protected area	4 versus 1	4	205.3	0.99

**Fig 4:**
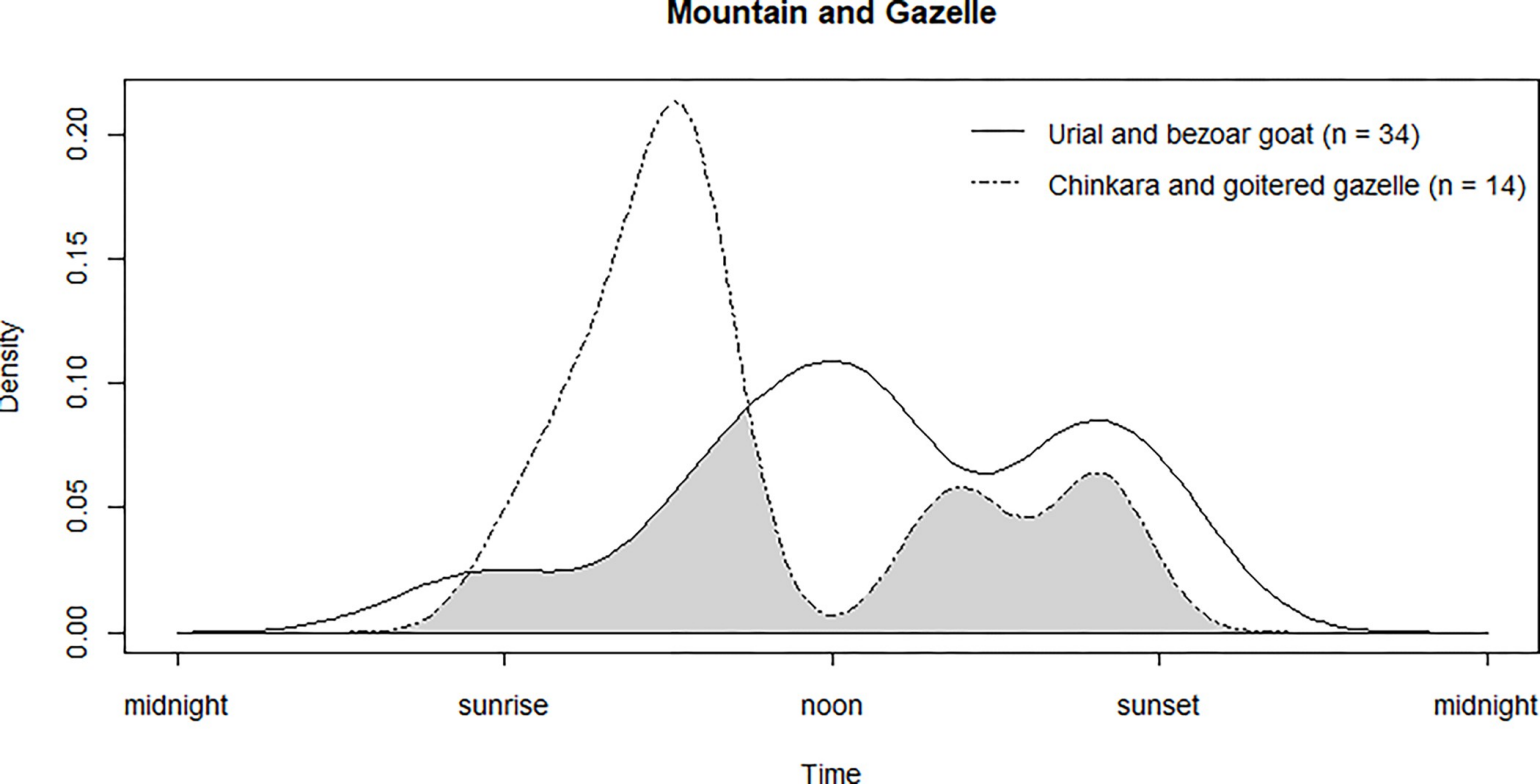
Temporal patterns of Asiatic cheetah hunting efforts for two types of prey, mountain ungulates such as urial and bezoar goat versus plain-dwelling ungulates such as goitered gazelle and chinkara, based on opportunistic sightings (2000–2021). Gray areas underneath density curves represent the overlapped area.

The coefficient of overlapping indicated a moderate degree of overlap (Δ_1_ = 0.57 [CI = 0.30–0.74]) between the timing of hunting for two different types of prey as mountain ungulates (i.e., urial and bezoar goat) versus plain-dwelling ungulates (i.e., goitered gazelle and chinkara; Fig 4); however, there was no evidence for differences in the activity curves between their temporal pattern (*U*^2^ = 0.24, *P* = 0.19). Nonetheless, it seemed that hunting gazelles mainly occurred after sunrise whereas mountain ungulates were the main prey between noon and sunset (Fig 4).

## Discussion

### Historical foraging ecology of Asiatic cheetahs

Ethnobiological and historical records suggested that gazelle species were the main prey for cheetahs across their Asian range. Nonetheless, gazelles were never the exclusive prey for Asiatic cheetahs. Conversely, other prey species, notably medium-sized ungulates living in hilly and mountain landscapes were commonly reported to be preyed upon by Asiatic cheetahs since historical times. In contrary to our first hypothesis, we therefore conclude that the predation on mountain medium-sized ungulates is not an emerging hunting behavior in Asiatic cheetahs.

Our study showed that the prey species for cheetahs highlighted in zoological records largely corresponded to the existing composition of ungulates in each country. Medium and large-sized ungulates inhabiting drylands were recorded as the cheetah prey. In Africa, cheetahs are generally considered to capture medium-sized prey [61–63] within a preferred body mass range of 23–56 kg [61]. However, their accessible prey, i.e., prey in the weight ranges preferred and killed relative to their abundance, varies between 14 and 135 kg [62], showing their wide spectrum of prey.

As ungulates with the largest distribution, gazelles were widely reported as the main prey for Asiatic cheetahs before their widespread extirpation in the 20^th^ century [5, 6, 41, 42, 44, 47, 49, 50]. However, when sympatric, mountain ungulates were also reported as cheetah prey such as urial in the USSR, Turkmenistan, Kazakhstan [6] and Iran [12, 45]. In the rest of Asiatic cheetah range, particularly across the Arabian Peninsula, the only sympatric medium sized ungulates were gazelles, which provided a narrow prey base for Asiatic cheetahs.

### Recent foraging ecology of Asiatic cheetahs

Extensive faecal sampling between 2006 and 2017 ranked urial as the main prey base of cheetahs (46.6% FO) while the collective contribution of two species of gazelles, i.e., chinkara and goitered gazelle did not exceed a small proportion (13.2% FO). Similarly, in 55.2% (n = 69) of records of opportunistic sightings of cheetah hunting made by wildlife conservation rangers between 2000 and 2020, the urial was the major prey while the contribution of the two gazelle species was 20% (n = 25) in sighted hunting efforts. All these two sources of evidence confirmed that the urial is currently the main prey for Asiatic cheetahs in Iran. Given the recent scarcity of gazelle species in west and central Asian countries [7, 17, 64, 65], cheetah predation on urials most likely has increased in recent times. However, the lack of observational records or faecal samples from the pre-1970s period to compare with the recent records require us to view our findings about the possible larger contribution of urial in recent times as suggestive rather than conclusive.

Nonetheless, our review of published studies investigating the faecal samples of Asiatic cheetahs in Iran suggested that the contribution of gazelles to the cheetah diet was likely overestimated in historical records. Where multiple prey was present for cheetahs in Iran in recent times, they mainly fed on urial and bezoar goat, even when gazelles were present [2] while it would be expected for cheetahs to hunt gazelles if they were their main prey. This foraging pattern based on recent data along with multiple records of urials in historical and ethnobiological records suggested that the actual contribution of mountain ungulates to the Asiatic cheetah diet has been underestimated [12, 15]. The 12^th^ century *Baznameh Nasavi* reported that cheetahs move to colder environments, i.e. near mountains, in summers [56]. Similarly, Northwest African cheetahs (*A*. *j*. *hecki*) prey on a spectrum of medium-sized ungulates including gazelles and Barbary sheep (*Ammotragus lervia*), but they often stayed close to massifs and mountains in summers [66]. Hilly and mountain landscapes mainly associate with higher water availability, and consequently in montane desert habitats, cheetahs probably had greater hunting success [67–69]. Reviewing historical zoological records as well as recent faecal analysis studies showed that the contribution of domestic animals to the dietary requirements of Asiatic cheetahs is minor. In fact, we did not find any historical records noting livestock depredation by cheetahs in Asia. In contrast, human-cheetah conflict is a major conservation concern for the persistence of cheetahs in many parts of Africa [70, 71]. Nonetheless, the minor role for domestic stock consumption recorded in previous studies based on faecal sampling may also be an underestimate [2, 4, 58–60], not reflecting stock predation and defecation in excursions beyond the protected area boundaries when wild prey is depleted, and large number of livestock are available [37], particularly in Asiatic cheetahs which regularly wander beyond the protected area boundaries [69, 72, 73]. Retaliation by local people following cheetah depredation on their livestock have resulted in multiple casualties for cheetahs [20]. The remaining population of Asiatic cheetahs in Iran is too small to sustain any additional mortalities, and therefore mitigation efforts are urgently needed, particularly outside the protected areas where the risk is maximized [20].

### Characteristics of prey choice in Asiatic cheetahs

We could not find any evidence of the effect of social structure, season, or protected area on the prey choice in Asiatic cheetahs, contrary to our second hypothesis [29, 30]. Bezoar goats are the largest medium-sized ungulates in Iran, often found in rugged, precipitous terrains [74] which are less suitable as habitats of cheetahs [75–77]. We expected that bezoar goats would be preyed upon by cheetah coalitions, which corroborate those of studies done in Africa which found that male coalitions killed larger prey species [30, 78]. More than half of the kills made by coalitions in Iran were bezoar goats; however, our sample size for kills that were made by coalitions was too small to test its significance.

Cheetah populations are known to show some degree of specialization in their choice of prey [30, 78] or hunting habitat [31, 79]. Our work revealed selective hunting of adult male (> 4 years) urials and bezoar goats, which is in accordance with previous observations of prey sex selectivity by cheetahs in central Iran [80] as well as Africa [63, 81] Antipredator vigilance, which is more common in females and their young among mountain wild sheep and goats [37, 82], is a possible explanation for apparent male-selective predation by Asiatic cheetahs. Given the similar male-skewed predation by other large carnivores in west and central Asia, such as grey wolf *Canis lupus* [83] and leopard *Panthera pardus* [37, 84], understanding the effects of multi-predator predation on recruitment and survival of ungulates, particularly in areas with running trophy hunting is recommended [85].

Although hunting attempts for different prey types, i.e., plain-dwelling gazelles versus mountain ungulates, showed high temporal overlap, it also showed that most of the gazelle hunting occurred in mornings while the predation on mountain ungulates was predominantly between mid-day and evening. This behavior can be explained in two ways. First, gazelles show a bimodal feeding activity during crepuscular peaks [86, 87], which overlaps with cheetahs hunting peaks in the mornings [88]. Similarly, mountain ungulates exhibit the same activity budget, but generally with a daily altitudinal movement, spending overnight in high altitudes while grazing at lower hills during daytime [89]. The latter period is when they are most susceptible to cheetah predation, which often happens midday onwards when they are in their lower areas. Second, unlike the general contention that cheetahs have shifted their habitat to higher elevations [2, 3, 19, 76, 77], It is unlikely that cheetahs hunt in rugged high elevation terrain, but more likely that they wait for their mountain prey to descend to lower altitudes, which is aligned with their sprint-based hunting strategy. In accordance with our third hypothesis, the persistence of Asiatic cheetahs based on multiple prey associated with different habitat types (open plain versus hilly and mountain landscapes) has been possible due to spatiotemporal plasticity in their foraging behavior.

There are two limitations to our study. First, although the ethnobiological records provided an insight into prey-predator interaction between cheetahs and their prey in historical times, the lack of data on prey availability in those records prevented us from inferring the prey selection of Asiatic cheetahs in historical times. Second, we obtained zoological records from the entire range of cheetahs in west and central Asia to illustrate the historical foraging ecology of cheetahs. Nonetheless, our ethnobiological records only represented Iran while the addition of this type of records from other countries within the range of Asiatic cheetahs, particularly Saudi Arabia, Turkmenistan, Pakistan and India, could have improved our understanding of the historical foraging ecology of Asiatic cheetahs at the continental scale.

Management implications Our findings highlighted three management implications to improve the restoration of Asiatic cheetahs:

1. Artificial provision of water and supplementary feed for wild ungulates is an ongoing management intervention in many cheetah protected areas in Iran, particularly in arid years [20]. However, the question is where to implement these management interventions? Our findings showed that developing water sources as well as the provision of supplementary feed should be established in transition areas between low and high elevations, notably hilly terrains to enable the cheetah to have access to the prey spectrum available in desert environments.
2. Asiatic cheetahs showed spatiotemporal plasticity in foraging behavior which enabled them to persist based on a wide range of prey species. Therefore, in contrary to the general perception that associated cheetahs with open plains as their main habitat [42–44, 50], it is likely that cheetahs persisted in more heterogenous landscapes based on the last documented records in gazelle-dwelling open areas in west and central Asia. Therefore, future reintroduction efforts for cheetahs across their former Asian range may find our study helpful in developing management plans in areas with multiple prey and heterogenous habitat types.
3. With the growing number of rewilding efforts across the world, particularly for megafauna [90], the use of ethnobiological records can effectively improve our knowledge about the ecological and evolutionary processes and outcomes of different species across wide range of time scales. Our study highlighted the importance of learning from the past in order to better plan and manage a species recovery in the present and future.

Despite the intensive conservation efforts of the last two decades, the Asiatic cheetah remains critically endangered and on the verge of extinction.

## Supporting information

S1 Table List of the zoological records in west and central Asian countries (1890–1980) which were investigated. We reviewed 30 records, but 11 were excluded because of the lack of acknowledgement of cheetah prey and habitat or language barrier (S1 Table). Therefore, 19 zoological records were included in the current study.
(XLSX)

S2 Table List of the historical books from Iran which were investigated. (XLSX)

S3 Table List of the ethnobiological records (1000–1900) from Iran which were investigated. (XLSX)

S4 Table List of published papers in peer-reviewed journals (n = 5) to obtain faecal samples (collected between 2006 and 2017) from six cheetah protected areas in Iran. (XLSX)

S5 Table Details of direct sighting of cheetah predation behavior in Iran (2000–2021), based on interviewing wildlife conservation rangers (n = 115) working within the cheetah protected areas to record any sighting of cheetah predation behavior. The data were assigned as “hunting”, i.e., the cheetah was seen while ambushing and chasing the prey or “foraging”, defined when the cheetah was seen eating the prey.
(XLSX)

S1 Data (XLSX)
